# Survey of predictive value of 4-hour urine collection for diagnosis of proteinuria in preeclampsia

**Published:** 2013-08

**Authors:** Maryam Asgharnia, Roya Faraji, Nooshaz Mirhaghjoo, Zahra Atrkar Roshan, Babak Ashrafkhani, Mina Moslehi

**Affiliations:** 1 *Reproductive Health Research Center, Department of Obstetrics and Gynecology, Guilan University of Medical Sciences, Rasht, Iran.*; 2 *Department of Midwifery, Guilan University of Medical Sciences, Rasht, Iran.*; 3 *Department of Biostatistic, Guilan University of Medical Sciences, Rasht, Iran.*; 4 *Guilan University of Medical Sciences, Rasht, Iran *

**Keywords:** *Preeclampsia*, *Proteinuria*, *Gestationalhypertension*

## Abstract

**Background:** Measuring the 24-hour urine protein ≥300 mg is the standard threshold value for diagnosis of preeclampsia.

**Objective:** This study was intended to determine if a patient’s 4-hour urine protein correlate with the 24-hour value for diagnosis of preeclampsia.

**Materials and Methods:** This was a cross sectional study performed on 84 women with suspected preeclampsia due to positive urinary test strip with minimum protein content of 1+ and BP ≥140/90 at Al-zahra Educational Hospital in Rasht (Iran) from May 2007 to January 2008. Urine samples were collected within 24 hours in successive periods: The first 4-hour and the next 20-hours urine, in separate containers. The protein contents of 4-hour and 24-hour urine samples were calculated. Data were analyzed by intra-class correlation coefficient, and Receiver Operating Characteristic (ROC) curve.

**Results:** The ROC curve showed the cut-off point of 55.5 for 4-hour urine protein. The correlation between 4- and 24-hour urine protein excretions identified that most women (about 85.1%) with protein excretion rate of 300 mg/24h or more (with preeclampsia) had the same amount of protein of 55.5 or more in their 4-hour urine excretion (p<0.001). Also, most of them (about 83.7%) with a total urinary protein excretion of less than 300 mg/24h (no preeclampsia) had a protein excretion rate of less than 55.5 mg/4h.

**Conclusion:** This study showed 4-hour protein collection can be used as acceptable substitute for assessing the protein content of 24-hour urine samples as a more convenient, faster, and cheaper method for diagnosis of preeclampsia and the cut-off point for 4-hour urine protein is 55.5 mg.

This article extracted from a submitted thesis. (Mina Moslehi)

## Introduction

Hypertensive disorders are among the most prevalent medical complications of pregnancy (1). Approximately 70% of these disorders are gestational hypertension, sometimes called pregnancy-induced hypertension (2). Preeclampsia which is included in this category is identified with hypertension and proteinuria (3, 4). Studies indicate that the presence of both proteinuria and hypertension during pregnancy can significantly increase the risk of maternal complications and prenatal mortality (5). Some studies, also, confirm preeclampsia as the main cause of 15% of preterm deliveries and 16% of maternal mortality (6, 7). 

Hypertensive disorders of pregnancy, and particularly preeclampsia, are also common in Iran and are considered as one of the major causes of premature birth, maternal mortality, and morbidity (8). Although hypertension is an important factor in the diagnosis of preeclampsia, it is strongly suggested to consider the presence and degree of proteinuria as another essential criterion to assure the diagnosis. Proteinuria is an important objective diagnostic criterion, the more severe the proteinuria and the more certain is the diagnosis of preeclampsia (5, 9). Proteinuria is defined as the urinary excretion of 300 mg of protein or more in a 24-hour urine collection or the excretion of 30 mg/dl of protein, in a random urine sample (10). 

Using only one random urine sample might not be adequate in assessment of significant proteinuria (5, 11-13). Therefore, a precise detection of the rate of urine protein is possible only through measuring the 24-hour urine protein excretion and, hence, a 24-hour urine analysis has been considered as the gold standard. 

Recent studies have shown incomplete 24-hour urine collections among 37% of the samples (14). In addition, one of its major disadvantages is the duration of the time period required to collect the patient’s urine. The 24-hour period required for the collection of urine often results in a delay in the diagnosis and treatment, or the prolongation of hospital stay (10, 15). So, it is necessary to find a faster and simpler method. Studies show little or no change in the amount of protein being excreted in 24-hour urine in hospitalized women on bed rest (16, 17). 

Therefore, it is possible to use samples collected in shorter time for measurement of urinary protein and a 4-hour urine protein measurement can be practiced as an alternative diagnostic method for evaluation of proteinuria in women with preeclampsia. The results of the study done by Wongkitisophon *et al*, on 38 pregnant women diagnosed with hypertensive disorders of pregnancy, indicate that the 4-hour values of urine protein correlate with those of 24-hour samples in women with pregnancy hypertension (18). 

Also, the results in a similar study done by Shahgheibi *et al* on 58 pregnant women diagnosed with hypertensive disorders suggest that protein concentration in 4-hour urine samples positively correlates with the protein concentration in 24-hour samples (19). Although the correlation between 4 and 24-hour urine protein values was examined in previous studies, the cut-off point of protein in 4-hour urine collection for predicting proteinuria was not evaluated. Also, the number of the patients in the previous studies was limited. Therefore, in order to get a practical use out of the results, the present study was intended to determine the 4-hour urine collection values for the detection of proteinuria in women with preeclampsia who referred to Alzahra Center. 

Based on the findings, we can measure cut-off point of 4-hour urinary protein in order to use it as an alternative method in patients with preeclampsia which eventually minimizes the risks of maternal-fetal complications through shortening the period required for the diagnosis of preeclampsia and making quicker medical decisions.

## Materials and methods

This analytic cross sectional study was conducted on pregnant women <20 weeks of gestation with suspected preeclampsia who were referred to Al-Zahra Educational Hospital in Rasht, Iran from May 2007 to January 2008. In this study, all hypertensive patients with more than 20 weeks of gestation, a blood pressure of ≥140/90, and a positive proteinuria (at least 1+) were included. 

The participants signed an informed consent document before joining the study. Finally a total of 84 women were found to be eligible for this study. Women were excluded if they had a clinical indication for delivery at the time of admission (symptom for severe preeclampsia). Also exclusion criteria included severe preeclampsia, chronic hypertension before pregnancy or before the 20^th^ week of pregnancy, disorders such as chronic renal diseases, diabetes, lupus, and urinary tract infection. Moreover, those who, for any reason, could not have complete or accurate 24-hour urine collection were not included in this study. 

The patients were on modified bed rest and the urine collection started on the first morning after admission to the hospital. Before the urine collection, all women were cautiously instructed on the procedure. Besides, a two-part questionnaire consisting of demographic and urine analysis data of patients was developed. In this study, 3 various urine samples were collected: the random urine samples for diagnosis, the first 4-hour urine sample (from 8 am to 12 noon) and the subsequent 20-hour urine sample. The first step was to measure the protein and creatinine concentration in the first 4-hour urine volume. Then, by adding the 20-hour urine sample, we obtained the 24-hour urine volume which was measured for the second time (the total 24-hour urine value was calculated by adding up the urine samples in the two containers). 

The urine protein and creatinine were calculated in 4-h, 24-hour and random urine sample collections by the laboratory technician in hospital using Pyrogallol Red Method. Proteinuria was defined as a 24-hour urinary protein excretion of more than 300 mg. The random urine samples were examined to identify proteinuria and the results were reported as negative, trace, +1, +2, +3 and +4. The approval letter was obtained from Guilan University of Medical Sciences Ethic Committee with 1910231221 number. This study was done with financial support of Vice chancellor of research Guilan University of Medical Sciences.

**Statistical analysis**

Data analysis was performed using SPSS software (Statistical Package for Social Sciences, Version 16). The relationship between protein-to-creatinine ratios in the 4- and 24-hour urine samples was measured using the intra-class correlation coefficient. The 24-hour urine protein was used as a gold standard to determine the sensitivity, specificity, and positive and negative predictive values of 4-hour urine sample. The Receiver Operating Characteristic (ROC) curve was used to determine the cut-off point for predicting proteinuria.

## Results

**Figure 1:**
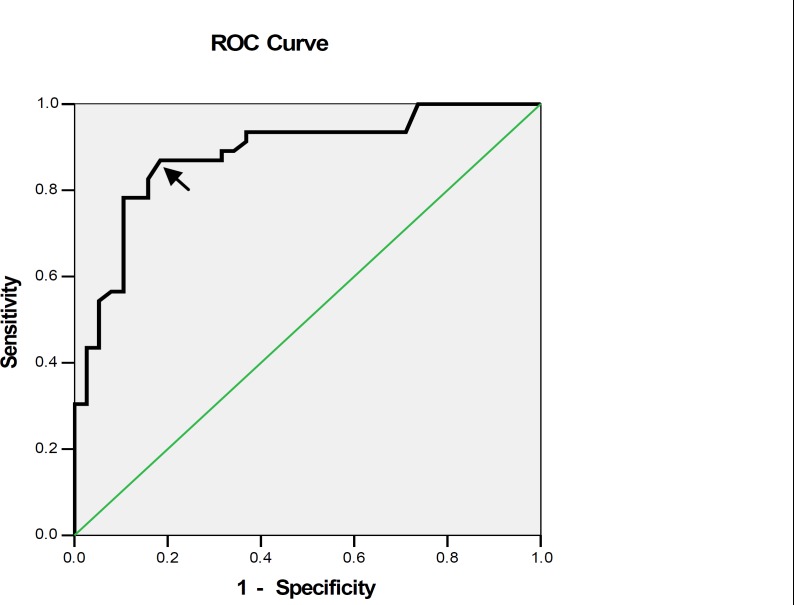
Predictive values of 4-hour urinary protein excretion for detection of significant proteinuria with various cut offs. The ROC curve showed the cut-off point of 55.5 for 4-hour urine protein.

The findings indicate that the mean age of the studied population was 30.4±6.43 years and their mean gestational age was 32.65±4.04 weeks. 57.1%were nuliparous and the mean urinary protein excretion in 4- and 24-houe urine collections were 164.76 and 1001.45 mg respectively. The ROC curve showed the cut-off point of 55.5mg for 4-hour urine protein. The correlation between 4- and 24-hour urine protein excretions identified that most women (about 85.1%) with protein excretion rate of ≥300mg /24h (who were diagnosed with preeclampsia) had the same amount of protein of 55.5 or more in their 4-h urine excretion (positive predictive value). Also, most of them (about 83.7%) with a total urinary protein excretion of less than 300mg /24h (who had no preeclampsia) had a protein excretion rate of less than 55.5 mg/4h (negative predictive value) (Figure 1). 

The area ROC curve identified that a value of 55.5mg in the 4-hour urine sample has the highest prediction value, (in comparison with 24-hour protein measurement). The 4-houre urine protein results correlated positively with the 24-hour results for diagnosis of preeclampsia (r=0.9, p<0.001). The positive and negative predictive values were 85.6% and 83.7% respectively with a sensitivity and specificity of 86.9% and 81.5% in 4 hour urine protein excretion. By the ROC curve analysis, the cut-off points of 0.20 for 4-hour urine P/C ratio and 0.21 for random urine P/C ratio were identified as the best threshold to predict proteinuria (≥300 mg) in the studied population. 

Because the average of the random and 24-hour urine P/C ratio in our study showed statistically significant difference, an interclass correlation coefficient was calculated to determine the correlation between 4- and 24-hour urinary protein-to-creatinine ratios. The obtained result of 0.9 indicates an excellent agreement between 4- and 24-hour P/C ratio and the correlation coefficient of 0.83 indicates an excellent agreement between random and 24-hour urine P/C ratio.

## Discussion

The gold standard for proteinuria and a key component in the assessment of preeclampsia is 24-hour urine collection. But it is time-consuming and cumbersome both for the patients and the staff handling the urine collection. An accurate substitution of a 4-h urine collection, in terms of specificity and sensitivity, would be an easier test for the patients to take; it does not include lab and collection errors happening in the measurement of protein excretion in 24-hour urine and causes facilitation of diagnosis and prompt clinical decision making as well (18).

The present study is intended to determine the correlation between the results of the measurement of protein excretion in 4-h and 24-hour urine collection. The results indicate that a cut-off point of 55.5 mg and more, which corresponds with a protein excretion rate of ≥300 mg/24h is predictive of preeclampsia. 

The study demonstrated a significant correlation between protein-to-creatinine ratio in 4- and 24-hour urine collections (p<0.0001, r=0.90) and like the studies conducted by Rodriguez-Thompson, Wheeler, and Huang there was a strong correlation between the P/C ratio in 4-h urine samples and urinary protein excretion in 24-hour collections (12, 20, 21). In our study, the sensitivity and specificity of 4-h protein excretion were 86.9% respectively, with the positive and negative predictive values of 85.1% and 83.7%.

Whereas in the study conducted by Sikul, in which the P/C ratio of 4-h urine collection was considered as an accurate and gold standard in women with preeclampsia, sensitivity, specificity, positive predictive value and negative predictive value were 81%, 88%, 93% and 71% respectively (22). Several investigations have explored proteinuria in a shorter period. 

Amirabi *et al* divided their patients into three groups for proteinuria (no proteinuria, mild and severe proteinuria), they showed that value of 4-h sample period did correlate with that of 24-hour samples for mild and severe proteinuria, with a significant correlation between 4- and 24-hour urine protein concentration (p<0.001, r=0.97), in which sensitivity and specificity of 4-h urine test were 93.2% and 90.2% respectively (23). Also Rabiee indicated protein value for the first 8 or 12=h of 24-hour urine samples correlated with 24-hour samples for patients with proteinuria (24). Our results were similar to these studies. 

In recent study there was a high correlation of 12-h urine protein >165 mg with a 24-hour urine protein ≥300 mg. That was also with the benefit of a shorter evaluation time (25). The difference of findings between their report and ours may be due to the difference in the study population, the method of urine collection and the test method. The wider exclusion criteria in our population may justify the higher positive and negative predictive values found in our study. For example in Adelberg *et al* study patients with hypertensive disorders of pregnancy were included and the urine was collected in first 8 hours, next 4 hours and remaining 12 hours urine sample. Total protein values for 8 and 12-hour urine samples correlate positively with values for 24-hour for patients with proteinuria. In their study the 8 and 12 hour urine samples collected in different times of the day but in our study we collect urine samples from 8 a.m. to 12 noon and then the subsequent 20-hour urine sample. Although they didn’t show significant pattern of diurnal variation in protein excretion and 8 and 12 hour urine results were collected at different times of the day, which correlated with day 24 hour value for patients with mild and severe proteinuria (26).

One of the advantages of the present study is obtaining random urine specimens from women with suspected preeclampsia before taking 24-hour urine collection. Furthermore, the patients with the history of diabetes, high blood pressure and lupus nephritis were excluded from the study to avoid the influence of renal dysfunction on the results. However, patients with severe preeclampsia were excluded because of delivery prior to collection of samples and the need for urgent termination and it was not possible to include severe preeclampsia in our study. 

These were some of the limitations and concerns associated with this study. Based on the findings of the present study, we conclude that the measurement of protein and protein-to-creatinine ratio in 4-h urine samples could be a reasonable alternative test for the detection of proteinuria in the patients with preeclampsia when it is not easily possible to collect 24-hour urine samples. Further investigations with more samples are suggested to prove the accuracy of the study.

## Conflict of interest

There is no conflict of interest in this work.
